# Thermally Triggered Vanishing Bulk Polyoxymethylene for Transient Electronics

**DOI:** 10.1038/s41598-019-54565-5

**Published:** 2019-12-02

**Authors:** Dongqing Liu, Songhe Zhang, Haifeng Cheng, Renfu Peng, Zhijian Luo

**Affiliations:** Science and Technology on Advanced Ceramic Fibers and Composites Laboratory, National University of Defense Technology, Changsha, 410073 P.R. China

**Keywords:** Electronic devices, Polymer characterization

## Abstract

Transient materials capable of disappearing rapidly and completely are critical for transient electronics. End-capped polyoxymethylene (POM) has excellent mechanical properties and thermal stability. However, research concerning the inherent thermal instability of POM without end-capping to obtain transient rather than stable materials, has never been reported. Here, POM without end-capping is proposed as a novel thermally triggered transient solid material that can vanish rapidly by undergoing conversion to a volatile gas, and a chemical vapor deposition method is developed to obtain a smooth POM substrate from the synthesized POM powder. Experimental and theoretical analysis was employed to reveal the mechanism whereby the POM substrate formed and vanished. A Cr/Au/SiO_2_/Cu memristor device, which was successfully deposited on the POM substrate by physical vapor deposition, exhibits bipolar resistive switching, suggesting that the POM substrate is suitable for use in electrical devices. Thermal triggering causes the POM substrate to vanish as the memristor disintegrates, confirming excellent transient performance. The deposited bulk POM material can completely vanish by thermally triggered depolymerization, and is suitable for physically transient substrates and packaging materials, demonstrating great prospects for application in transient electronics for information security.

## Introduction

Transient electronic devices, which physically disappear or undergo structural fragmentation over a specified period of time after a period of stable usage^1–3^, are of great interest in various applications ranging from biomedical implants to environmental protection and information security^4–6^. The development of physically transient materials is critical for the advancement of transient electronics and their applications. As reported thus far, a large number of transient materials, including metals^7^, oxides^3^, nitrides^8^, small molecule organic materials^9,10^, soluble polymers^11^, and depolymerizable polymers^12–15^, have been exploited. Among these materials, depolymerizable polymers that respond to a specific stimulus by unzipping into small molecules are crucial to recent advances in the field. For example, light-sensitive poly(phthalaldehyde) in conjunction with photoacid generators was fabricated and employed as substrate material for transient electronics^16^. Unfortunately, the integration of photoacid generators complicates the preparation process and usually produces a large amount of residue. Depolymerizable polymers with a low ceiling temperature (*T*_c_), which enables direct and rapid vanishing in response to a thermal triggering, have been proposed as potential material for encapsulating or forming the substrate material for transient electronics recently^17,18^. However, a low *T*_c_ yields low thermal stability and easy depolymerization, which complicates the processing of a polymer into bulk material for device deposition, thereby limiting its practical application as a heat-sensitive transient material^17,18^. Even if bulk polymer is obtained by the addition of a plasticizer, a large amount of residue usually remains after decomposition of the bulk material^18^. Therefore, transient materials with a large bulk that disappear by changing from a solid to a volatile gas with minimal or non-traceable remains are highly desirable in transient electronics.

**Figure 1:**
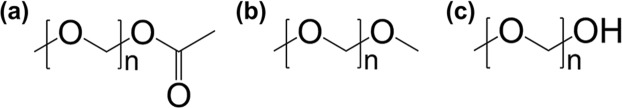
The molecular structure of POM (**a**) by esterification end-capping treatment, (**b**) without end-capping treatment and (**c**) with hydroxyl groups.

Polyoxymethylene (POM), a depolymerizable polymer, was first synthesized in 1859 by Butlerov^19^. However, it has not found practical application owing to rapid depolymerization caused by its low *T*_c_ of 119 °C^20,21^. In the late 1950s, DuPont demonstrated that anionic polymerization of formaldehyde followed by esterification end-capping (Fig. 1a) greatly enhances the thermal stability, thereby triggering a rapid increase in POM research^22–25^. Subsequently, alternative methods to stabilize POM, such as copolymerization, have been constantly proposed and improved upon^26^, achieving excellent mechanical properties, high chemical resistance to most solvents, and thermal stability^27–29^. Currently, POM is garnering extensive interest in the automotive industry, and also in the mechanical, electrical, and electronic industries^22^. Its development paths indicate that research pertaining to POM involves a continuous process of stabilization to improve its thermal stability, and focuses on developing enduring systems that are mechanically and thermally robust. However, the inherent thermal instability of POM obtained without end-capping, which depolymerizes rapidly to change from a solid to a volatile oxymethylene gas in response to thermal stimuli, has not been exploited in the past. Combined with the requirements for transient materials, the thermal instability of POM without end-capping inspired us to explore the possibility of using it as transient material because depolymerization converts POM into a volatile oxymethylene gas^30,31^.

Here, we propose the use of POM as a novel thermally triggered transient material to satisfy current transient requirements. We exploit the ability of POM, to depolymerize rapidly to undergo conversion from a solid to a volatile gas without leaving a residue when exposed to sufficient heat. In addition, we provide a method based on chemical vapor deposition (CVD) to prepare a POM substrate from the synthesized POM powder. Experimental and theoretical analysis was employed to reveal the mechanism by which the POM substrate formed and vanished. Then, a Cr/Au/SiO_2_/Cu memristor device was successfully deposited on the POM substrate. This device exhibited bipolar resistive switching, suggesting that the POM substrate is suitable for use as an electrical device. Apart from this, the POM/Cr/Au/SiO_2_/Cu memristor device disintegrated when heated, triggered by the vanishing POM substrate, to show excellent transient performance. This work opens the window to utilizing the thermal instability of POM with potential application to transient electronics.

## Experimental

### Synthesis of POM

Trioxymethylene (10 g) was added to cyclohexane (20 mL) at 80 °C. After complete dissolution, the solution was cooled to 40 °C. Then, boron trifluoride diethyl etherate (0.1 mL) as initiator was injected into the trioxymethylene solution, which was stirred for 1 h at 40 °C. The product was collected by filtration and washed four times using acetone.

### Thermally triggered experiment of POM in a closed system

A certain amount of POM powder was placed in a 25-mL round-bottomed flask and the neck of the flask was fitted with a balloon before the flask was heated in an oil bath at 170 °C until the POM powder at the bottom of the flask had completely vanished.

### Preparation of bulk POM substrate

The procedure of the CVD method that was developed to obtain bulk POM substrate is as follows. The synthesized POM (2 g) was placed in the bottom of a 50-mL beaker, and the substrate was placed on the beaker. A hot stage was heated to 250 °C, after which the beaker was placed on the hot stage to start deposition. The POM at the bottom of the beaker evaporated completely and the substrate was removed. After cooling for 5 minutes, the bulk POM was lifted from the substrate.

### Preparation of Au/SiO_2_/Cu memristor

A layer of Cr (20 nm thick), which served as an adhesive layer, was deposited on the POM bulk by electron-beam evaporation (Kurt J Lesker Co., USA). Then, the 100-nm-thick Au BE, followed by a layer of SiO_2_ (20 nm thick) for resistive switching, and the 50 nm thick Cu top electrode (TE) were deposited with a stencil mask.

### Characterization

Fourier transform infrared spectroscopy (FT-IR) measurements were acquired using a Thermo Scientific Nicolet iS 50 spectrometer. Thermogravimetric-differential scanning calorimetry (TG-DSC) analysis was conducted on a TA Instruments SDT Q600 using a heat ramp of 10 °C/min under air atmosphere. The molecular weight-distribution was measured on a Waters 1515 gel permeation chromatography (GPC) with hexafluoroisopropanol (HFIP) as solvent. Scanning electron microscopy (SEM) was captured on a Hitachi S4800 and the accelerating voltage was 1.0 kV. Atomic force microscopy (AFM) was carried out on an NT-MDT Solver with scanning frequency of 1 Hz. The current-voltage (*I*-*V*) curve of the Au/SiO_2_/Cu memristor was tested using a Keithley 4200 semiconductor characterization system with a Cascade Microtech Summit 11000 probe station. The transient characteristics were studied by placing the samples on a hot stage for accurate temperature control and heated until the desired trigger temperatures were reached. Depolymerization processes were recorded by using a Canon E400 camera.

## Results and Discussion

**Figure 2:**
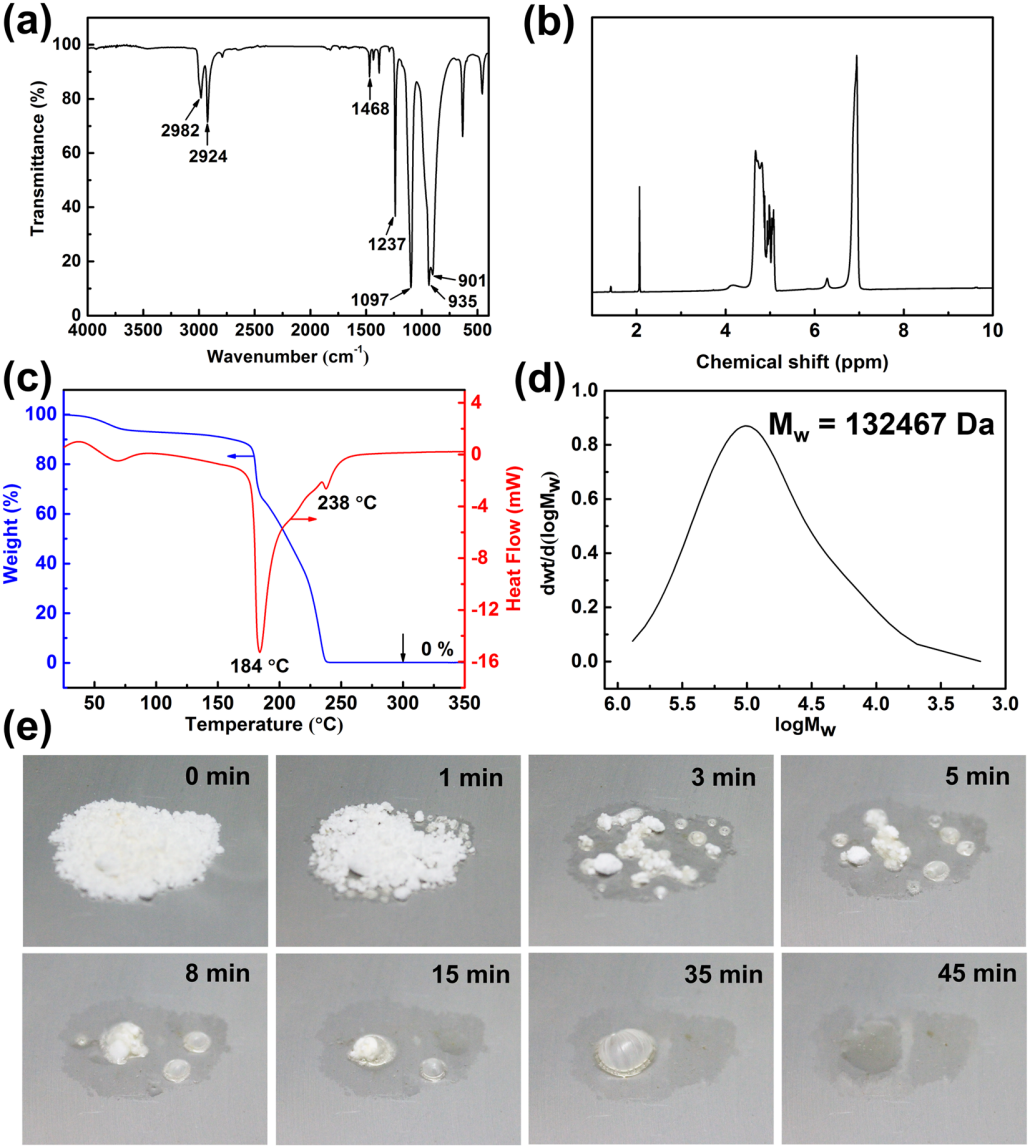
Characterization of POM: (**a**) FT-IR spectrum of POM powder. (**b**) ^1^H NMR spectrum of POM powder dissolved in HFIP at room temperature, and then mixed with deuterated acetone in a 1:1 volume ratio. (**c**) TG-DSC curves of POM powder in air atmosphere using a heat ramp of 10 °C/min. (**d**) Molecular weight-distribution curve of POM powder dissolved in HFIP at 35 °C (*M*_w_ 132,467 Da; high polymerization degree of approximately 4414). (**e**) Optical images recorded during vanishing of about 0.1 g POM at various times after thermal triggering at 180 °C.

POM was synthesized by cationic ring-opening polymerization using trioxymethylene as the starting agent and the Lewis acid boron trifluoride diethyl etherate as the initiator (Fig. S1, Supporting Information). FT-IR revealed the typical structure of POM, as shown in Fig. 2a, including the stretching vibration absorption (2982 cm^−1^ and 2924 cm^−1^), bending vibration absorption (1468 cm^−1^) of CH_2_, and vigorous coupling reaction vibrations (1237 cm^−1^, 1097 cm^−1^, 935 cm^−1^, and 901 cm^−1^) among the adjacent ether bonds in the POM backbone, thereby confirming the successful preparation of POM^24^. The ^1^H nuclear magnetic resonance (NMR) spectrum shown in Fig. 2b also demonstrated that the molecular structure of POM contains CH_2_ and CH_3_. The molecular structure of the synthesized POM powder without end-capping treatment is shown in Fig. 1b.

The results of the TG-DSC analysis are presented in Fig. 2c and show the thermal decomposition process of the POM powder. The TG curve indicates that POM undergoes decomposition in the temperature range 150–240 °C, and the mass loss is 100%. The existence of two endothermic peaks on the DSC curve indicates that the thermal decomposition of POM occurs in two stages. The endothermic peak at 184 °C corresponds to the melting temperature of POM, and the other peak at 238 °C is attributed to the thermal degradation temperature of POM. Figure 2d shows the molecular weight-distribution curve of POM powder. A solution of POM was prepared with HFIP as solvent at 35 °C. This solution was used to determine the weight average molecular weight (*M*_w_) characterized by GPC as 132,467 Da. According to the *M*_w_ and molecular structure of POM powder, a high degree of polymerization of approximately 4,414 was calculated. Instead of preparing thermally stable POM by esterification end-capping (Fig. 1a) as in previous research^23–25^, we prepared POM without end-capping treatment to achieve outstanding thermal instability. Figure 2e exhibits a set of optical images at various times recorded during the vanishing process of the POM powder weighing roughly 0.1 g after thermal triggering at 180 °C. The sample of POM powder vanishes rapidly within approximately 45 minutes at 180 °C, suggesting that POM powder prepared without end-capping possesses excellent thermal instability. As the triggering temperature is higher than the *T*_c_ (119 °C), close to the melting temperature (184 °C), and lower than the thermal degradation temperature (238 °C), thermal oxidative random scission followed by depolymerization is the main mechanism of vanishing^26,32^. Noteworthy is that the rate of decomposition and amount of POM powder remaining can be controlled by changing the thermally triggered temperature: higher temperature induces faster depolymerization and less residue (Fig. S2, Supporting Information).

Although POM without end-capping is specifically thermally unstable, the synthesized powder cannot be used directly; thus, it is necessary to fabricate the material in bulk. Traditional processing techniques for polymers such as hot pressing, injection molding, and melt extrusion^18,22^, require the material to be heated to temperatures above its melting temperature, which is limited to POM without end-capping. Solvent casting is another means of processing polymers, but the poor solubility of POM caused by its rigid crystalline structure even in the reportedly optimal solvent HFIP at room temperature precludes film formation^33^. If POM without end-capping is useful for transient electronics, the key would be to develop a suitable method for POM powder molding. First, a pressed-disk technique that does not require heating and dissolution is proposed to harvest the POM substrate (Fig. S3, Supporting Information). The decomposition behavior of the POM substrate is analogous to that of pristine POM material on account of their identical composition. However, large surface corrugations that might be induced by surface squeezing resulting from interaction between the sample and the mold, are visualized and generate a root-mean-square (RMS) roughness of 591.5 nm (Fig. S4, Supporting Information). The high roughness makes POM prepared using the pressed-disk technique unsuitable for use as a substrate.

Then, a thermally triggered experiment in which POM powder is placed in a closed system motivated us to develop a new strategy for forming bulk POM. The balloon tied at the neck of the flask shows a certain degree of bulging (Fig. S5a, Supporting Information), presenting direct evidence that POM can be depolymerized to undergo conversion from the solid to the gaseous state by heating. Unexpectedly, a white sheet of bulk material was observed to be deposited in the upper part of the flask. Unlike the open system shown in Fig. 2d, the intermediate substance produced by the decomposition of POM vaporizes and cannot escape from the closed system, causing the gaseous product to deposit by desublimation when it comes into contact with the cold neck of the flask. A set of optical images that were captured during the decomposition of bulk POM exfoliated from the neck of the flask at various stages after thermal triggering is provided (Fig. S5b, Supporting Information). The bulk POM weighing roughly 0.05 g loses its original morphological characteristic after 3 minutes and eventually vanishes within 17 minutes.

**Figure 3:**
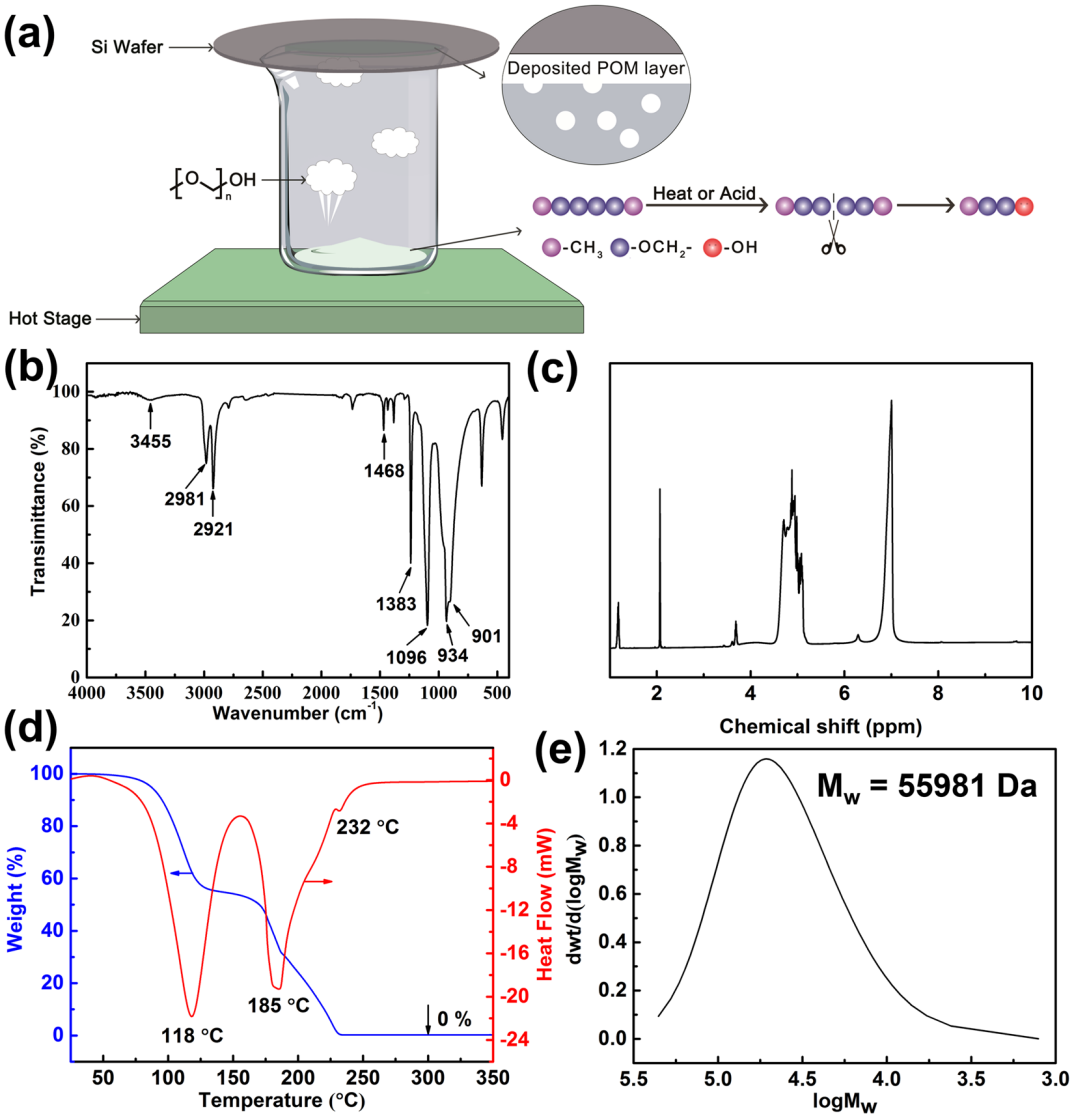
(**a**) Schematic diagram of the proposed CVD to obtain bulk POM substrate from POM powder. (**b**) FT-IR spectrum of bulk POM. The stretching vibration at 3455 cm^−1^ confirms the presence of hydroxyl in the molecular chain. (**c**) ^1^H NMR spectrum of POM bulk dissolved in HFIP at room temperature, and then mixed with deuterated acetone in a 1:1 volume ratio. (**d**) TG-DSC curves of bulk POM characterized in air atmosphere using a heat ramp of 10 °C/min. (**d**) Molecular weight-distribution curve of bulk POM dissolved in HFIP at 35 °C (*M*_w_ 55,981 Da; polymerization degree 1,865).

Inspired by the above unexpected experimental observation, we then attempted to use chemical vapor deposition (CVD) to obtain bulk POM substrate from POM powder. A schematic diagram of the proposed CVD is shown in Fig. 3a. Under heating, the intermediate **3** formed by the reaction of POM powder **1** with the action of heat or acid evaporates and is deposited on a cold Si wafer to form a bulk. Since POM is a temperature-sensitive transient material, the most important parameter of the CVD process associated with the POM film quality is the deposition temperature. Therefore, different deposition temperatures, such as 120 °C, 170 °C, 200 °C and 250 °C, were chosen to investigate the effect of temperature on the preparation of bulk POM. The results show that when deposition was carried out at 120 °C, only a mist-like film was produced on the substrate and no significant amount of solids were formed; When deposited at 170 °C, the formed sample had obvious delamination because of slow gas flow generation, and the sample was easily broken, making it difficult to peel off from the substrate. Bulk samples were obtained by CVD at both 200 °C and 250 °C. However, the surface roughness of the bulk POM obtained at 200 °C was larger than that obtained at 250 °C (Fig. S6, Supporting Information). A smooth substrate surface is required to ensure that the electrode is the electrically conductive. Therefore, 250 °C was chosen as the deposition temperature in this work. The formation mechanism of bulk POM was investigated by studying the polymer structure and decomposition process by using FT-IR, ^1^H NMR, TG-DSC, and GPC. The FT-IR spectrum in Fig. 3b shows that the absorption peaks of the bulk POM are consistent with that of pristine POM powder apart from the stretching vibration (3450 cm^−1^) of hydroxyl. In addition, the ^1^H NMR spectrum of POM bulk in Fig. 3c also shows the same chemical shift as POM powder apart from the chemical shift (3.69 ppm) of H in hydroxyl. That is, compared with POM powder, bulk POM contains terminal hydroxyl groups (Fig. 1c). As POM molecules with these terminal groups are readily depolymerized when the temperature is higher than its *T*_c_, the formation of hydroxyl groups enhances the thermal instability of the POM and improves the transient performance^33^. The TG curve shows that decomposition of the bulk POM occurs in the temperature range of 75–230 °C and the mass loss is 100% (Fig. 3d). The endothermic peak at 118 °C on the DSC curve (Fig. 3d) corresponds to the depolymerization temperature of the bulk POM with thermally unstable hydroxyl end-groups. The other peaks are attributed to the melting temperature and thermal degradation temperature of bulk POM at 185 °C and 232 °C, respectively. Figure 3e shows the molecular weight-distribution curve of the bulk POM. After the thermal decomposition process, the *M*_w_ of the POM was reduced from 132,467 Da to 55,981 Da. Because the molecular formula of the bulk POM can be expressed as CH_3_(OCH_2_)_n_OH, the degree of polymerization of approximately 1,865 is calculated. All these results indicate that the random scission reactions of the POM backbone that produce hydroxyl end-groups occur during the process of CVD.

**Figure 4:**
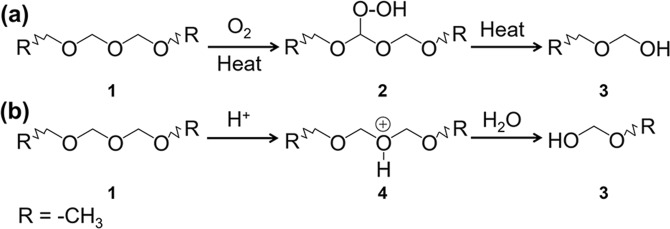
Inclusive scission reactions to produce terminal hydroxyl groups during the preparation process of bulk POM: (**a**) oxidative scission reaction and (**b**) acidolysis scission reaction. R represents the –CH_3_.

Previous research found that the oxidative scission reaction of POM starts at temperatures of approximately 160 °C and its origin is the formation of hydroperoxide groups in POM chains in the presence of oxygen^26^. Because CVD was carried out in an aerobic environment at 250 °C, which is in accordance with the conditions for oxidative scission reaction, we concluded that the oxidative scission reaction of POM powder occurs during CVD (Fig. 4a). With the action of heat and oxygen, intermediate **2** containing hydroperoxide groups is produced from POM powder **1**. Then, hydroperoxide groups induce *β*-scission of POM into fragments with hydroxyl end-groups **3**. In addition, because POM is sensitive to the acid formed by the oxidation of formaldehyde, the acidolysis scission reaction (Fig. 4b), which produces POM with terminal hydroxyl groups is also assumed to occur during CVD^34,35^. Subsequently, the generated POM with terminal hydroxyl groups **3** is partially vaporized by heat. The other part is depolymerized into formaldehyde gas, which will be expounded later. Because the substructure of POM containing the terminal hydroxyl groups cannot completely escape in our closed system setup, a deposit via desublimation is formed as it arrives at the cold Si wafer. This is where bulk POM is obtained.

**Figure 5:**
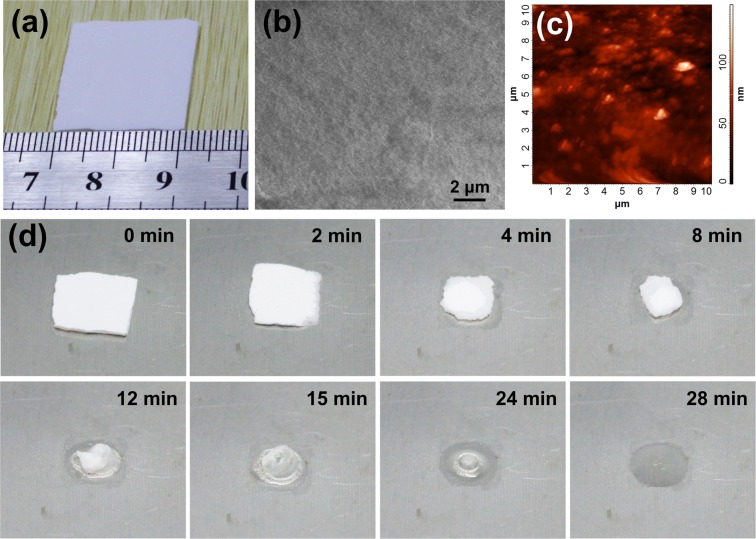
(**a**) Optical image of the bulk POM obtained by CVD. (**b**) SEM and (**c**) AFM images of the bulk POM. The POM is compact and surface smooth with a RMS roughness of 16.7 nm. (**d**) Set of images of bulk POM as a function of time after thermal triggering at 180 °C.

Images of the bulk POM obtained by CVD are shown in Figs. 5a and S7 (Supporting Information). The obtained POM bulk is flat and can be clamped. To investigate its suitability for use as transient substrate, SEM and AFM were performed. The results show that bulk POM is compact with a smooth surface (Fig. 5b) and an RMS roughness of 16.7 nm (Fig. 5c). To verify its transient property, thermal triggering experiment at 180 °C was conducted. Figure 5d shows a set of images recorded at various stages during decomposition of a 0.1 g bulk POM sample with an approximate size of 10 mm × 10 mm × 1 mm. It was observed that the volume of POM decreased and its complete disappearance is achieved within 28 minutes. These results suggest that bulk POM obtained via CVD is a promising candidate for use as a transient substrate material.

**Figure 6:**
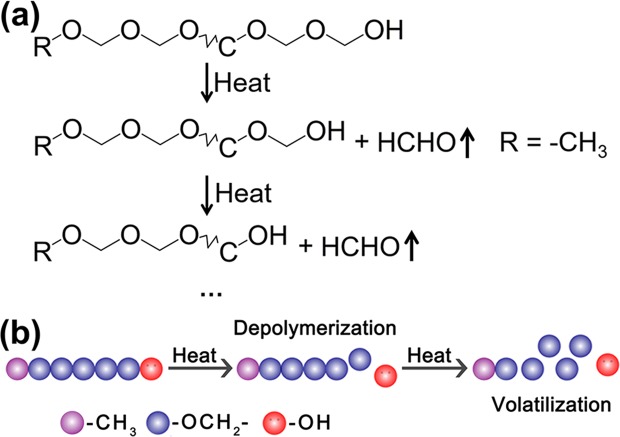
(**a**) Depolymerization of POM terminated with hydroxyl groups with continuous release of oxymethylene gas. R represents the –CH_3_. (**b**) The schematic of the POM vanishing.

Because the molecular structure of the bulk POM is terminated with hydroxyl groups, a reaction process shown in Fig. 6a responsible for POM vanishing after thermal triggering is proposed. When the bulk POM with unstable hydroxyl end group is heated, the active hydrogen in the hydroxyl group causes it to release oxymethylene gas while generating a new hydroxyl-containing POM. This step is followed and accompanied by a continuous release of oxymethylene until the complete unzipping of the macromolecular chain. The mechanism of POM vanishing is shown in Fig. 6b.

Bulk POM’s suitability as substrate and packaging for transient electronics was validated by investigating its hardness, surface roughness, and electrical insulation properties. A smooth surface is beneficial because it ensures the circuit conduction, whereas good hardness is advantageous for device processing and performance presentation. In addition, good electrical insulation is a key advantage to function as packaging. The Shore hardness and insulation resistance of bulk POM are approximately 20 HA and 1019 MΩ, respectively (Fig. S8, Supporting Information). All these results signify that bulk POM is promising as substrate and packaging applications.

**Figure 7:**
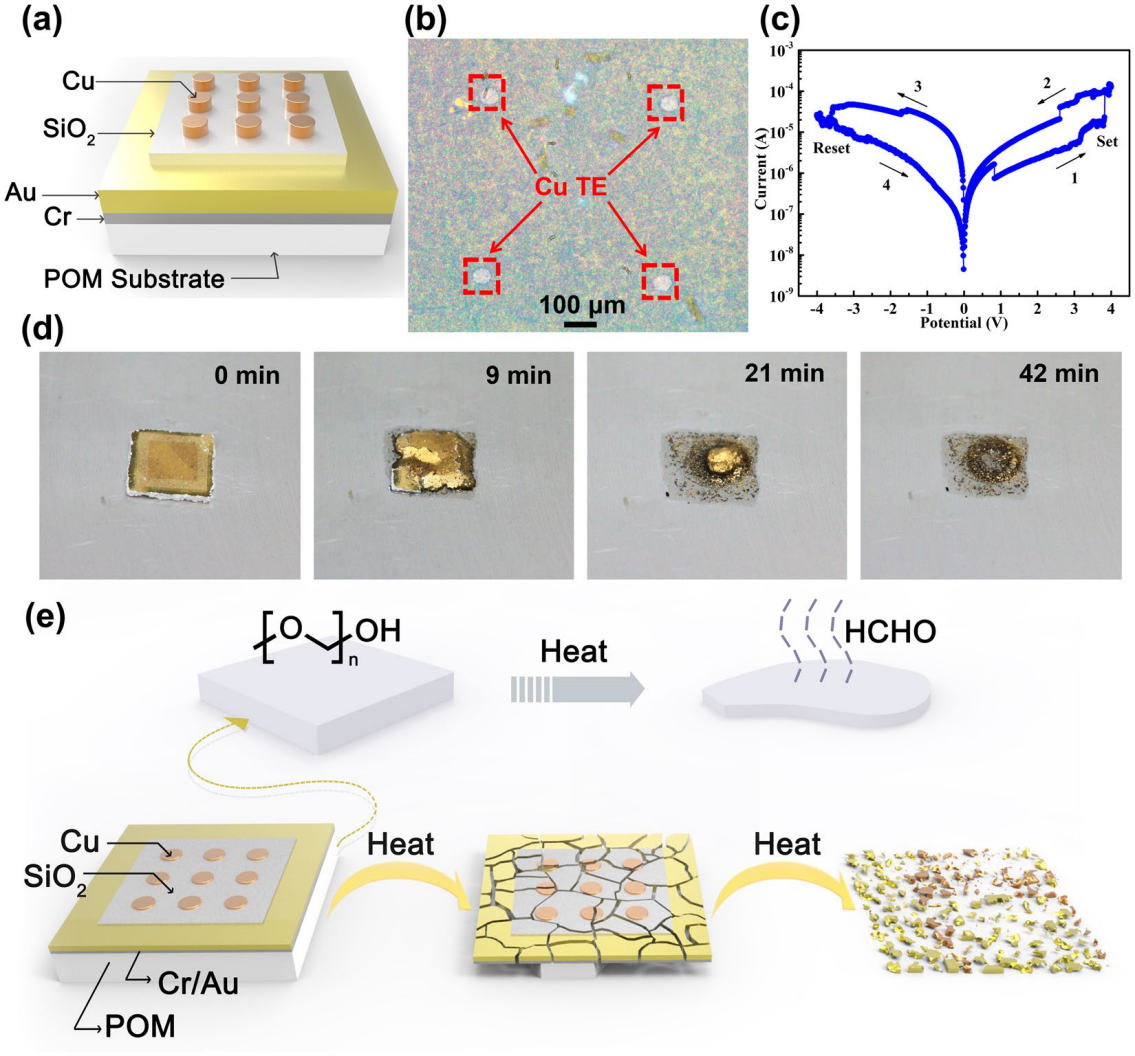
(**a**) Schematic diagram of Cr/Au/SiO_2_/Cu memristor on the POM substrate. (**b**) Optical image and (**c**) typical *I*-*V* characteristic of Cr(20 nm)/Au(100 nm)/SiO_2_(20 nm)/Cu(50 nm) memristor device on the POM substrate. Bias is applied to the Cu TE marked by red squares whereas the Au BE is grounded. (**d**) Set of images of the Cr(20 nm)/Au(100 nm)/SiO_2_(20 nm)/Cu(50 nm) memristor device on the POM substrate as a function of time with thermal triggering at 180 °C. The size of the POM substrate is approximately 10 mm × 10 mm × 1 mm. (**e**) Schematic diagram of POM/Cr/Au/SiO_2_/Cu device in the thermally triggered process.

A memristor, composed of an insulating material sandwiched between two conducting materials, is a fundamental circuit element with electrical properties. Therefore, it is an effective platform to fully examine the substrate can be used for electrical devices. Thus, a Cr(20 nm)/Au(100 nm)/SiO_2_(20 nm)/Cu(50 nm) memristor was prepared by electron-beam deposition on the POM substrate to further investigate the application potential of the POM substrate in transient electronics. Figure 7a shows a schematic diagram of the Cr/Au/SiO_2_/Cu memristor on POM substrate. Figure 7b shows an optical image of the Cr/Au/SiO_2_/Cu memristor on the POM substrate. The POM substrate was metallized by placing 20-nm thick Cr film and 100-nm thick Au film on the surface of the POM substrate to ensure good electrical conductivity. A typical current-voltage (*I*-*V*) characteristic with bipolar resistive switching of the Cr/Au/SiO_2_/Cu memristor was observed by imposing bias on the Cu TE, compatible with semiconductor technology and commonly used in memristors, and by grounding the Au bottom electrode (BE) (Fig. 7c). It functioned as expected with good steady state performance before a triggering condition is provided, suggesting that the POM substrate is suitable for use in electrical devices. The nonvolatile bipolar resistive switching behavior of devices can be attributed to the electrochemical formation and dissolution of conductive filaments in the dielectric layer^36^. Compared with the state-of-the-art memristors reported in literature^37,38^, its performance, including ratio of high resistance and low resistance and endurance of the Au/SiO_2_/Cu transient memristor was inferior. The main reason for this is that we only prepared a memristor to verify application potential of the POM substrate in transient electronics and performance optimization of the prepared memristor is already beyond the scope of our research.

Lastly, we demonstrated the transient behavior of an electronic device fitted with a vanishing POM substrate and a Cr/Au/SiO_2_/Cu memristor component. Figure 7d presents a set of images showing the changes in the Cr/Au/SiO_2_/Cu memristor device on a POM substrate with an approximate size of 10 mm × 10 mm × 1 mm as a function of time with thermal triggering at 180 °C. As POM substrate depolymerizes, the Cr/Au/SiO_2_/Cu memristor undergoes substantial structural collapse and disintegrates because of the vanishing POM underneath. Ultimately, complete destruction of the device is achieved within 42 minutes (Fig. S9, Supporting Information). Its electrical property after thermal triggering was also investigated to assess the transient characteristic. It was found out that the device was no longer conductive and lost the electrical property of the memristor after depolymerization for 8 min (Fig. S10, Supporting Information). Electrical performance testing for longer thermal trigger times has not been possible due to the fragmentation of the device into small debris. Although recently reported thermally triggered transient materials have a lower thermal triggering temperature^17,18^, it did not provide some evidence of the substrate demonstration in electronics. Moreover, a large amount of residue of the bulk substrate material can often be found. Figure 7e schematically illustrates typical stages of the transient process. The as-fabricated device shows the initially bipolar resistive switching. After thermal triggering, the POM substrate starts to depolymerize and releases volatile HCHO gas. Consequently, the device layer breaks into small debris because of the vanishing POM underneath, resulting to POM/Cr/Au/SiO_2_/Cu device failure. These results indicate that a POM substrate prepared by CVD can be used for transient electronics.

## Conclusions

A novel thermally triggered POM transient material based on the thermal instability of POM without end-capping was proposed. Its depolymerization only requires sufficient heat exposure. In addition, a simple CVD method was developed to obtain a compact smooth POM substrate. Moreover, the POM substrate is suitable for use in an electrical device and the POM/Cr/Au/SiO_2_/Cu memristor we fabricated shows excellent transient performance. The proposed POM material has great prospects for application in the field of transient electronics for information security.

## Supplementary information

Supporting Information

## Data Availability

The datasets generated and analyzed during the current study are available from the corresponding author on reasonable request.
