# Supplementary material for: Thermally Triggered Vanishing Bulk Polyoxymethylene for Transient Electronics
Source: Sci Rep. 2019 Dec 2;9:18107. doi: 10.1038/s41598-019-54565-5 (PMC6888842; doi:10.1038/s41598-019-54565-5)
Supplement: Supplementary file 1 — Supporting Information [file 41598_2019_54565_MOESM1_ESM.pdf]

Thermally Triggered Vanishing Bulk Polyoxymethylene for Transient Electronics

Dongqing Liu, Songhe Zhang, Haifeng Cheng, Renfu Peng, Zhijian Luo*

Science and Technology on Advanced Ceramic Fibers and Composites Laboratory, National University of Defense Technology, Changsha 410073, P. R. China
E-mail:liudongqing07@nudt.edu.cn

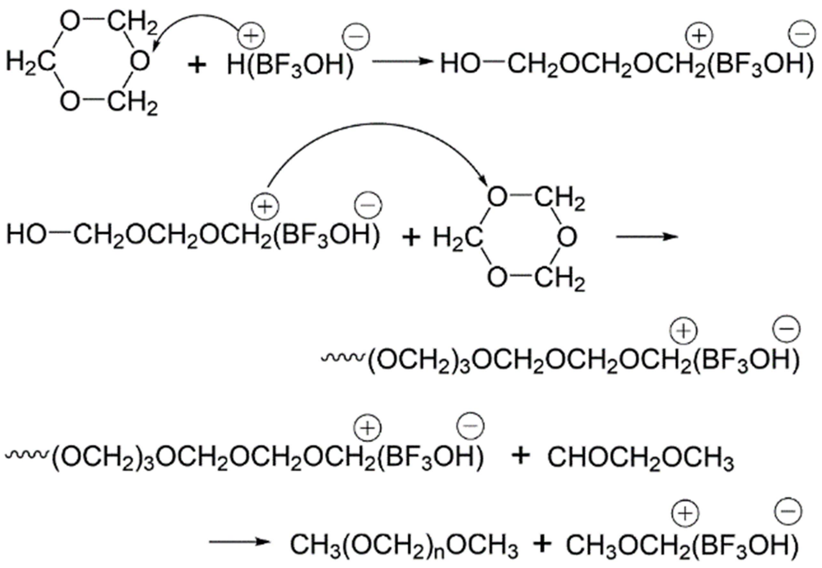

Figure S1. The synthetic process of POM by using cationic ring opening polymerization. The trioxymethylene uses as the starting agent and Lewis acid boron trifluoride diethyl etherate as the initiator.

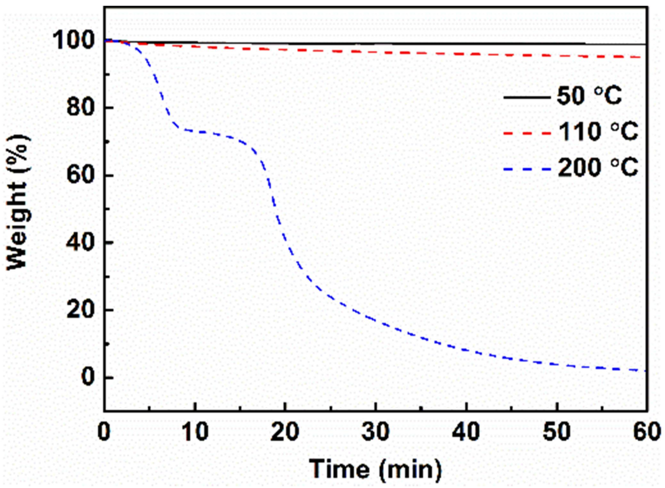

Figure S2. TG curves of POM powder at different temperature characterized in nitrogen atmosphere using a heat ramp of 10 °C/min.

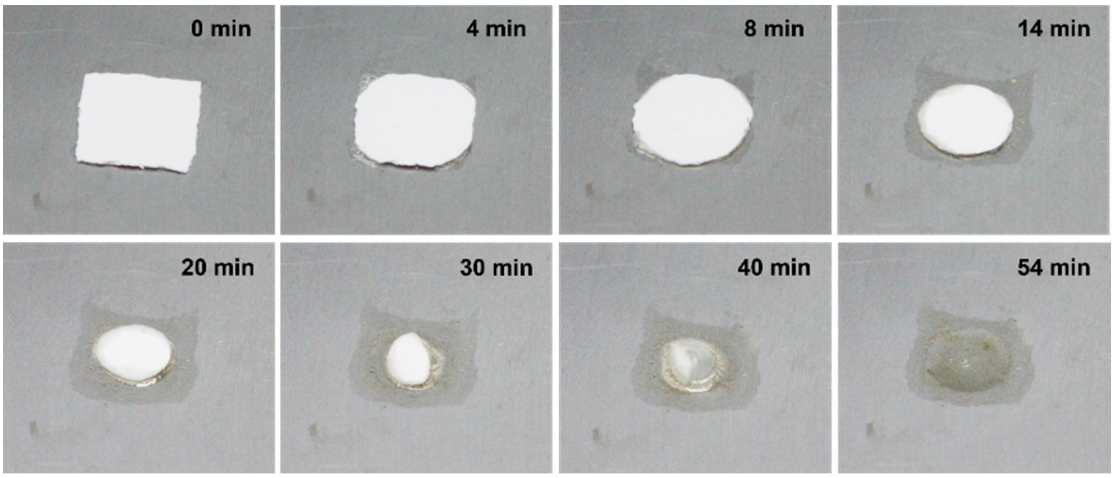

Figure S3. The optical images recorded during decomposition of POM prepared by pressed-disk technique at various time after thermal triggering at 180 °C. The applied pressure to prepare POM is 10 MPa, and the holding time is 30 seconds. The size of POM is about 10 mm×10 mm×0.5 mm.

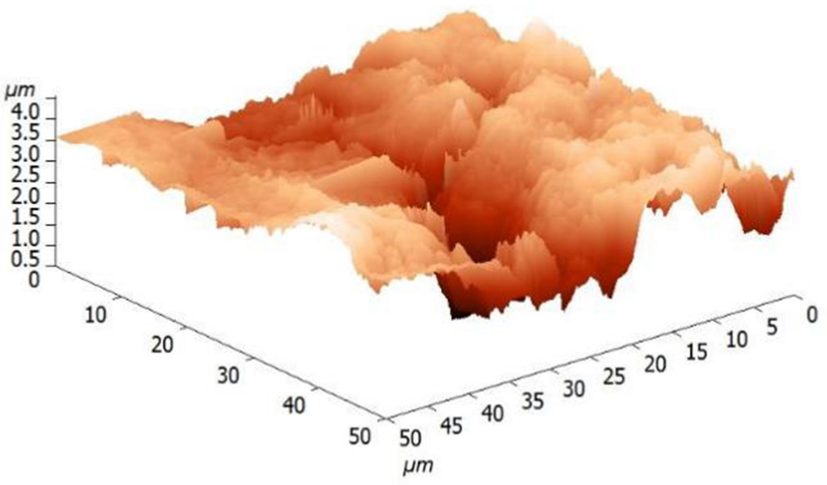

Figure S4. AFM image of POM prepared by pressed-disk technique. The RMS is 591.5 nm.

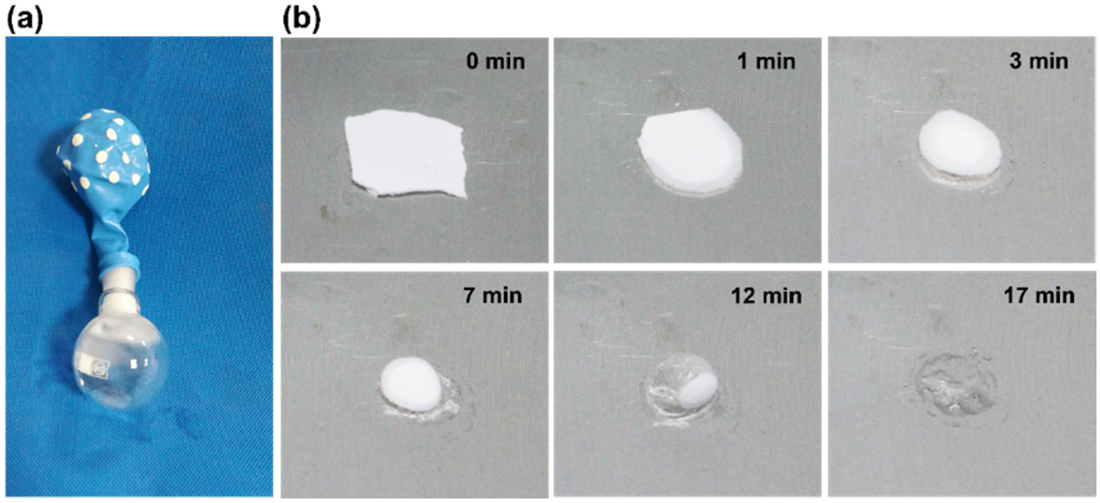

Figure S5. (a) The decomposition experiment of POM powder in a closed system to investigate the transient behavior of POM powder and observe the gas generation during the depolymerization. (b) The optical images during decomposition of POM exfoliated from bottle neck at various time after thermal triggering at 180 °C.

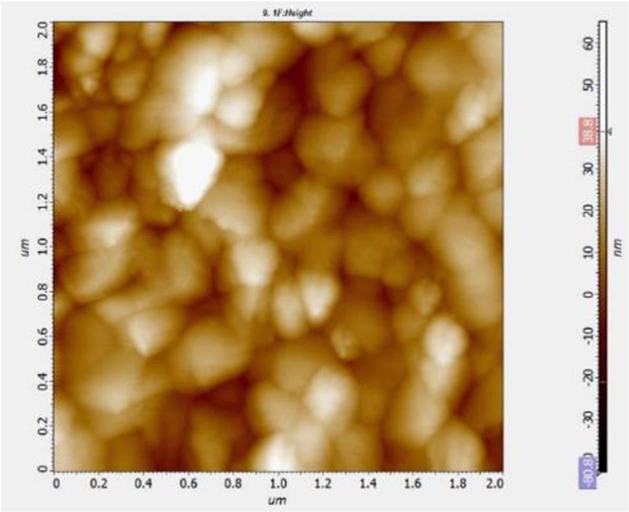

Figure S6. The AFM image of the bulk POM obtained at 200 °C.

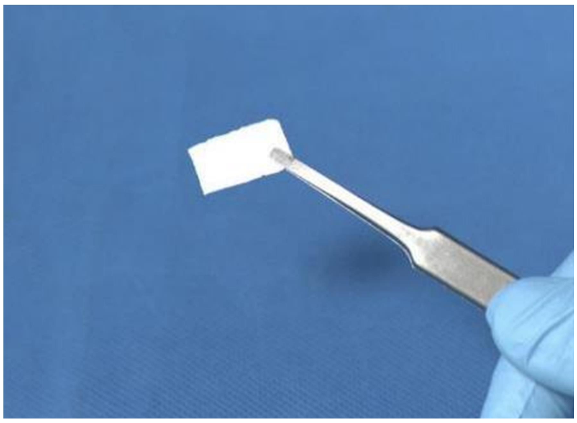

Figure S7. Optical image of the bulk POM.

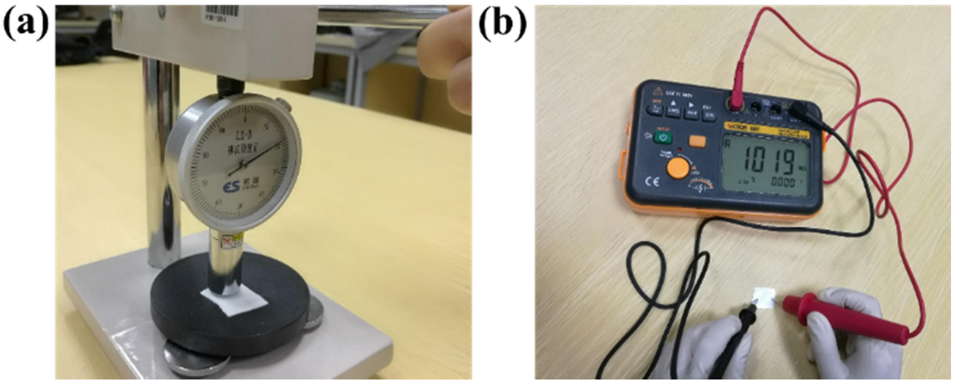

Figure S8. Shore hardness and electrical conductivity measurement of bulk POM. The Shore hardness and insulation resistance of bulk POM are approximately 20 HA and

1019 MΩ, respectively.

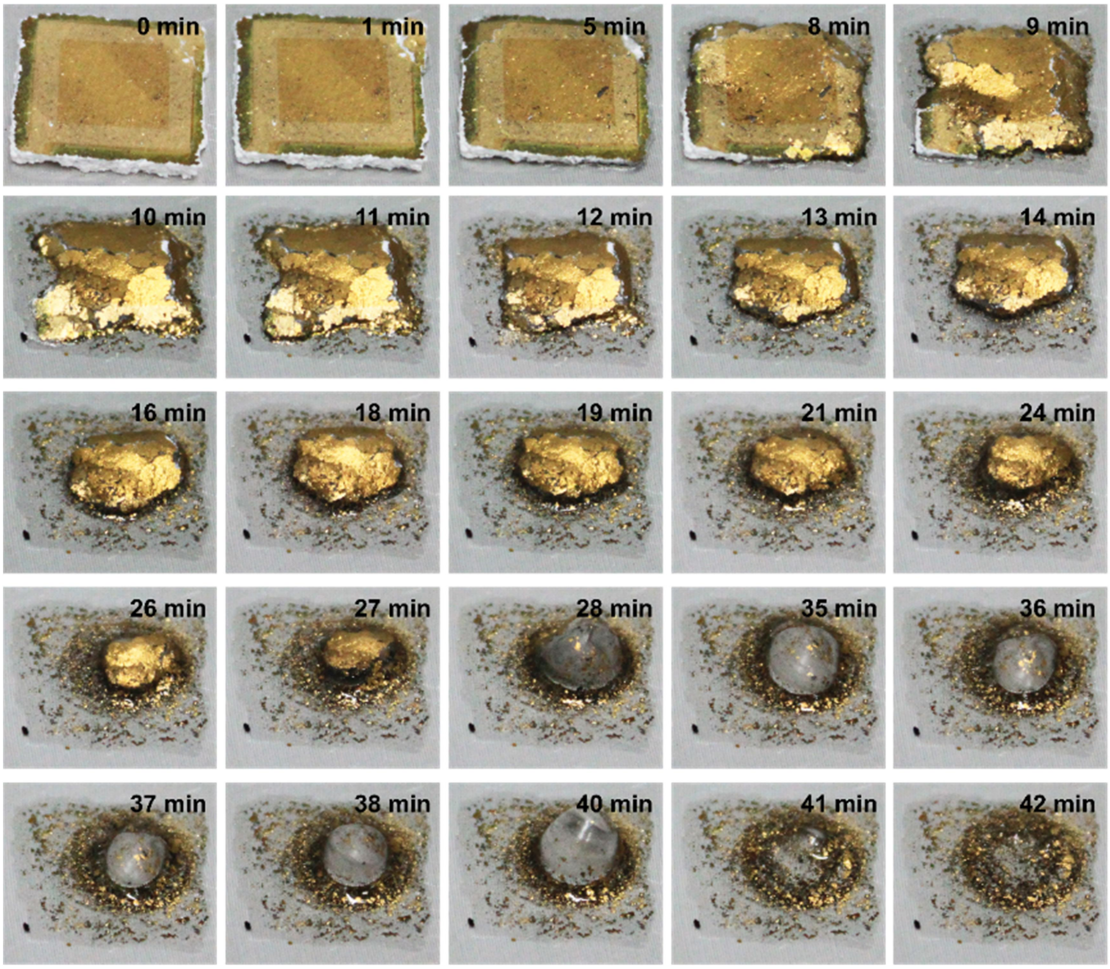

Figure S9. Set of images of the Cr(20 nm)/Au(100 nm)/SiO_2(20 nm)/Cu(50 nm) memristor device on the POM substrate as a function of time with thermal triggering at 180 °C. The size of the POM substrate is approximately 10 mm×10 mm×1 mm.

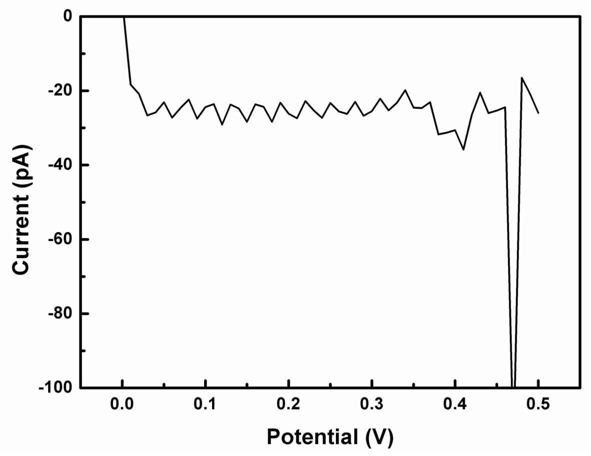

Figure S10. The electrical property of the memristor after thermal triggering for 8 min.
